# Supplementary material for: Structure of the human frataxin-bound iron-sulfur cluster assembly complex provides insight into its activation mechanism
Source: Nat Commun. 2019 May 17;10:2210. doi: 10.1038/s41467-019-09989-y (PMC6525205; doi:10.1038/s41467-019-09989-y)
Supplement: Supplementary file 1 — Supplementary Infomation [file 41467_2019_9989_MOESM1_ESM.pdf]

Structure of the human frataxin-bound Fe-S cluster assembly complex provides insight into its activation mechanism

N. G. Fox, X. Yu *et al*

SUPPLEMENTARY INFORMATION

Supplementary Table 1 Data collection, reconstruction, and model refinement statistics.

	SDAUF EMD-0560, PDB 6NZU	SDAUF EMD-0561
**Data collection**		
Microscope	Titan Krios	Titan Krios
Voltage (keV)	300	300
Nominal magnification	22,500 x	22,500 x
Exposure navigation	Stage Position	Stage Position
Electron exposure (e /Å^2)	42	42
Dose rate (e/pixel/sec)	5	5
Camera	K2 Summit	K2 Summit
Camera mode	Super-resolution	Super-resolution
Pixel size (Å)*	0.543	0.543
Defocus range (µm)	1.2 to 2.5	1.2 to 2.5
Micrographs Used	4260	4260
Final Refined particles (no.)	267,153	267,153
**Reconstruction**		
Symmetry imposed	C2	C1
Resolution (global)		
FSC 0.143	3.2 Å	3.4 Å
Applied B-factor (Å^2)	-178	-151
**Refinement**		
Protein residues	1592	
Ligands	6	
Map Correlation Coefficient	0.824	
R.m.s deviations		
Bond lengths (Å)	0.006	
Bond angles (°)	0.646	
Ramachandran		
Outliers	0.00 %	
Allowed	7.27 %	
Favored	92.73 %	
Poor rotamers (%)	0.00 %	
MolProbity score	1.45	
EMRinger score	3.98	
Clashscore (all atoms)	2.18	

*Calibrated pixel size at the detector

Supplementary Table 2 Proposed acyl-chain structures and relative abundances of acyl-ACP based on LC-MS.

m/z (4'-PP acyl chains)	elemental compositions*	predicted m/z	mass error (mDa)[#]	acyl structures[$]	acyl-chain length	relative abundances (%)[&]
443.295	$C_{23}H_{42}N_2O_4SH^+$	443.294	1.0	saturated acyl	C12	5.7
471.324	$C_{25}H_{46}N_2O_4SH^+$	471.326	-2.0	saturated acyl	C14	5.0
485.304	$C_{25}H_{44}N_2O_5SH^+$	485.305	-1.0	3-ketoacyl	C14	14.0
487.320	$C_{25}H_{46}N_2O_5SH^+$	487.321	-1.0	3-hydroxyacyl	C14	5.1
497.341	$C_{27}H_{48}N_2O_4SH^+$	497.341	0.0	2-enoylacyl	C16	15.5
499.358	$C_{27}H_{50}N_2O_4SH^+$	499.357	1.0	saturated acyl	C16	8.5
513.338	$C_{27}H_{48}N_2O_5SH^+$	513.336	2.0	3-ketoacyl	C16	22.6
525.372	$C_{29}H_{52}N_2O_4SH^+$	525.373	-1.0	2-enoylacyl	C18	14.9
541.369	$C_{29}H_{52}N_2O_5SH^+$	541.368	1.0	3-ketoacyl	C18	8.7

[*]Elemental compositions were derived based on accurate mass measurment.
[#]The mass errors are the differences between the experimental and predicted values expressed in mDa.
[$]Acyl-chain structures were proposed based on elemental compositions and the MS/MS/MS fragments.
[&]Relative abundances were derived based on extracted ion chromatograms of the 6+ holy-ACP molecular ions.

Supplementary Table 3 Primers used in this study.

Primer name	Primer
FXN_D124A_f001	GAGGACTATGcTGTCTCCTTTG
FXN_D124A_r001	AAACGTGTATGGCTTGTC
FXN_N146K_f001	ATGTGATCAAaAAGCAGACGC
FXN_N146K_r001	AGGTTCCTAGATCTCCAC
FXN_N151A_f001	GCAGACGCCAgcCAAGCAAATC
FXN_N151A_r001	TTGTTGATCACATAGGTTC
FXN_W155R_f001	CAAGCAAATCcGGCTATCTTCTC
FXN_W155R_r001	TTTGGCGTCTGCTTGTTG
FXN_P163G_f001	ATCCAGTGGAggTAAGCGTTATGACTG
FXN_P163G_r001	GGAGAAGATAGCCAGATTTG
FXN_R165C_f001	TGGACCTAAGtGTTATGACTG
FXN_R165C_r001	CTGGATGGAGAAGATAGC
ACPMfeI-f004	CTAGCTcaattgaTAAAGGAGG*UGG*ATGagcgacatgcctcctttgacgttag
ACP-XHO1-r001	CTAGCTctcgagTTAttcatatacatccttcttatc
To make the bi-cistronic cassette of His-ISD11 and NFS1	
LYRM4A-f201	TACTTCCAATCCATGGCAGCCTCCAGTCGCGCACAA
LYRM4A-r201	TTATAAATAAAATTATTTGTCAGGTCCTGGGCATGTCTCGAT
NFS1A-f202	AAATAATTTTATTTATAAGGAGATATACCATGCTGCGACCTCTCTATATGGAT
NFS1A-r201	TATCCACCTTTACTGTCAGTGTTGGGTCCACTTGATGC
To make the tri-cistronic cassette of His-ISD11, NFS1 and ISCU	
LYRM4A-f201	TACTTCCAATCCATGGCAGCCTCCAGTCGCGCACAA
LYRM4A-r201	TTATAAATAAAATTATTTGTCAGGTCCTGGGCATGTCTCGAT
NFS1A-f202	AAATAATTTTATTTATAAGGAGATATACCATGCTGCGACCTCTCTATATGGAT
NFS1A-rev	TTATTAAATAATATAATAGTCAGTGTTGGGTCCACTTGATGC
ISCUA-for	TATTATATTATTTAATAAGGAGATATACCATGTATCACAAGAAGGTTGTTGAT
ISCUA-r001	TATCCACCTTTACTGTCATTTCTTCTCTGCCTCTCCTT

Supplementary Table 4 SAXS Data collection and model refinement statistics.

Sample	SDAUF	SDAU
Structural Parameters		
Guinier		
I(0) (cm^3)	0.084	0.04
Rg (Å)	39.49	37
P(r)		
I(0) (cm^3)	0.084	0.04
Rg (Å)	39.87	37.16
Dmax (Å)	144	119
Shape model-fitting results		
DAMMIF	default parameters, 20 calculations	
q range for fitting	0.0095 – 0.2025	
Symmetry, anisotropy assumptions	P2, none	
χ^2 range	1.079 - 1.310	
Atomistic Modelling		
Model	SDAUF-Zn^{2+} cryo-EM structure	SDAU-Zn^{2+} structure (5WLW)
q range for all modelling	0.0075 – 0.3319	0.0081 – 0.3613
FoXS		
χ^2	1.98	1.11
Rg (Å)	38.56	36.51
c1,c2	0.99,-0.08	1.01,1.65

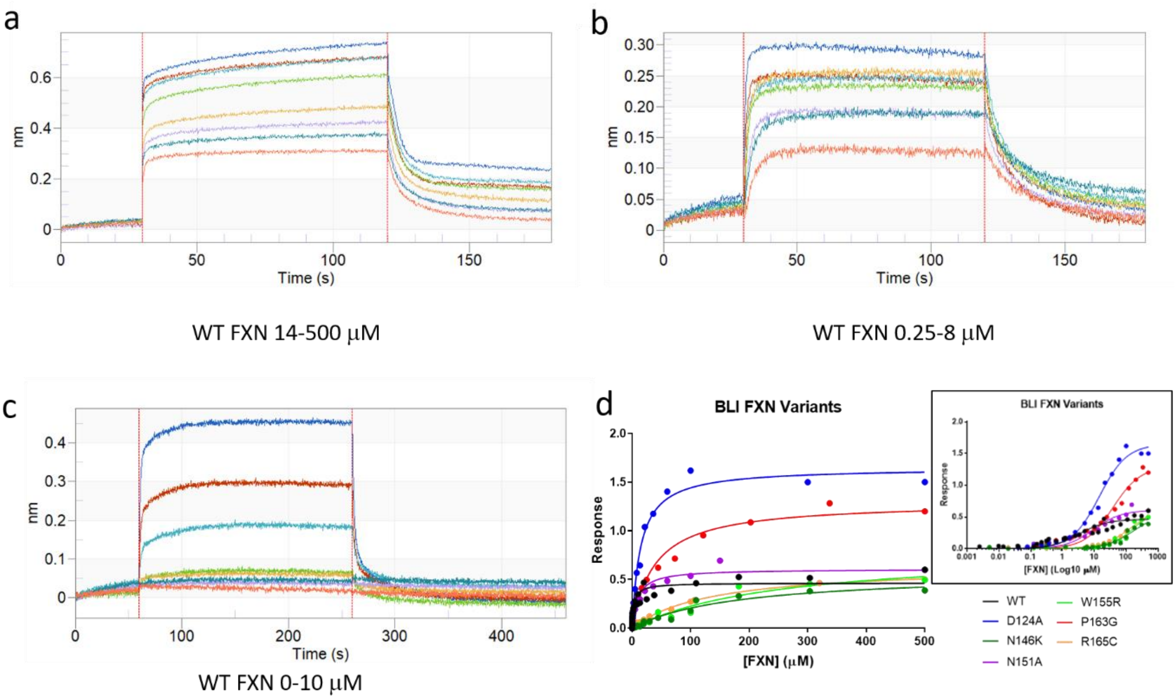

Supplementary Figure 1 Bio-layer interferometry of FXN WT and variants. Raw data curve for FXN(WT) binding to a biotinylated complex of ISCU with SDA_{ec} and titrating various concentrations of FXN ranging from 14-500 μM (**a**), 0.25-8 μM (**b**), and 0-10 μM (**c**). **d.** A plot of response vs. [FXN] (WT and variants) was used for K_d determination using one site-specific binding fit in GraphPad Prism (values reported in Fig. 2e). Inset shows a plot of response vs log_{10} [FXN] for clearer view of lower concentrations. Each data point was n=1 from a distinct sample of FXN made from serial dilutions. Source data are provided as a Source Data file.

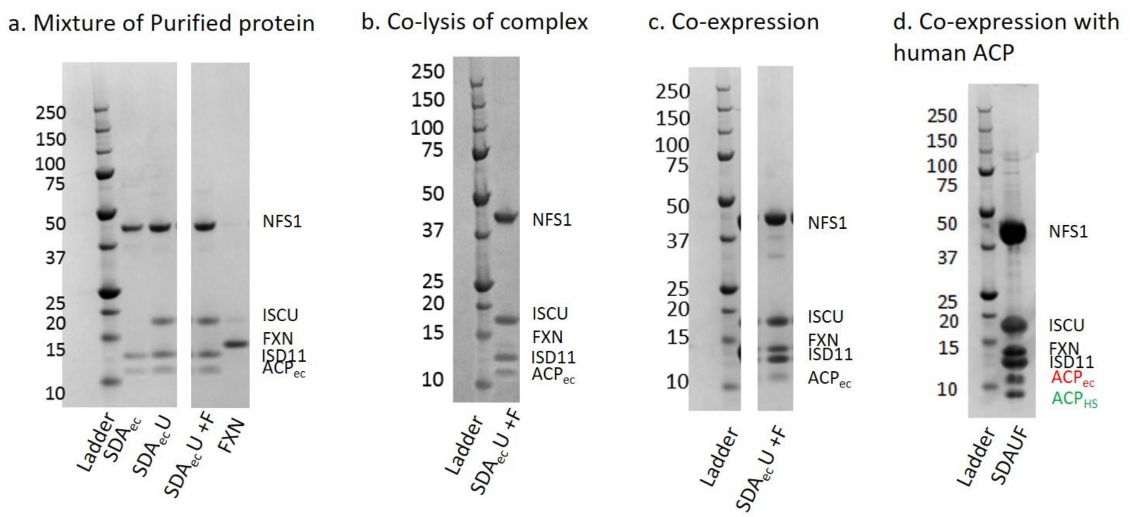

Supplementary Figure 2 Different attempts of purifying the SDAUF Complex. a. Pellets from the expression of the bi-cistronic plasmid (His-ISD11 and NFS1) and tri-cistronic plasmid (His-ISD11, NFS1, and ISCU) were purified and shown to co-purify with *E. coli* ACP (yielding SDA_{ec} and $SDA_{ec}U$, respectively). Purified FXN was added to purified $SDA_{ec}U$ complex and run on gel filtration where no FXN binding was observed ($SDA_{ec}U$ +F). **b.** Pellets from tri-cistronic expression and FXN expression were co-lysed and purified, showing very little FXN bound. **c.** Co-expression of tri-cistronic plasmid with FXN showed a 5-way complex of SDUF co-purifying with E. coli ACP. **d.** Co-expression of tri-cistronic plasmid with FXN and human ACP showed a 5-way complex of SDAUF with a mixture of both human and *E. coli* ACP. Full uncropped gels are provided as a Source Data file.

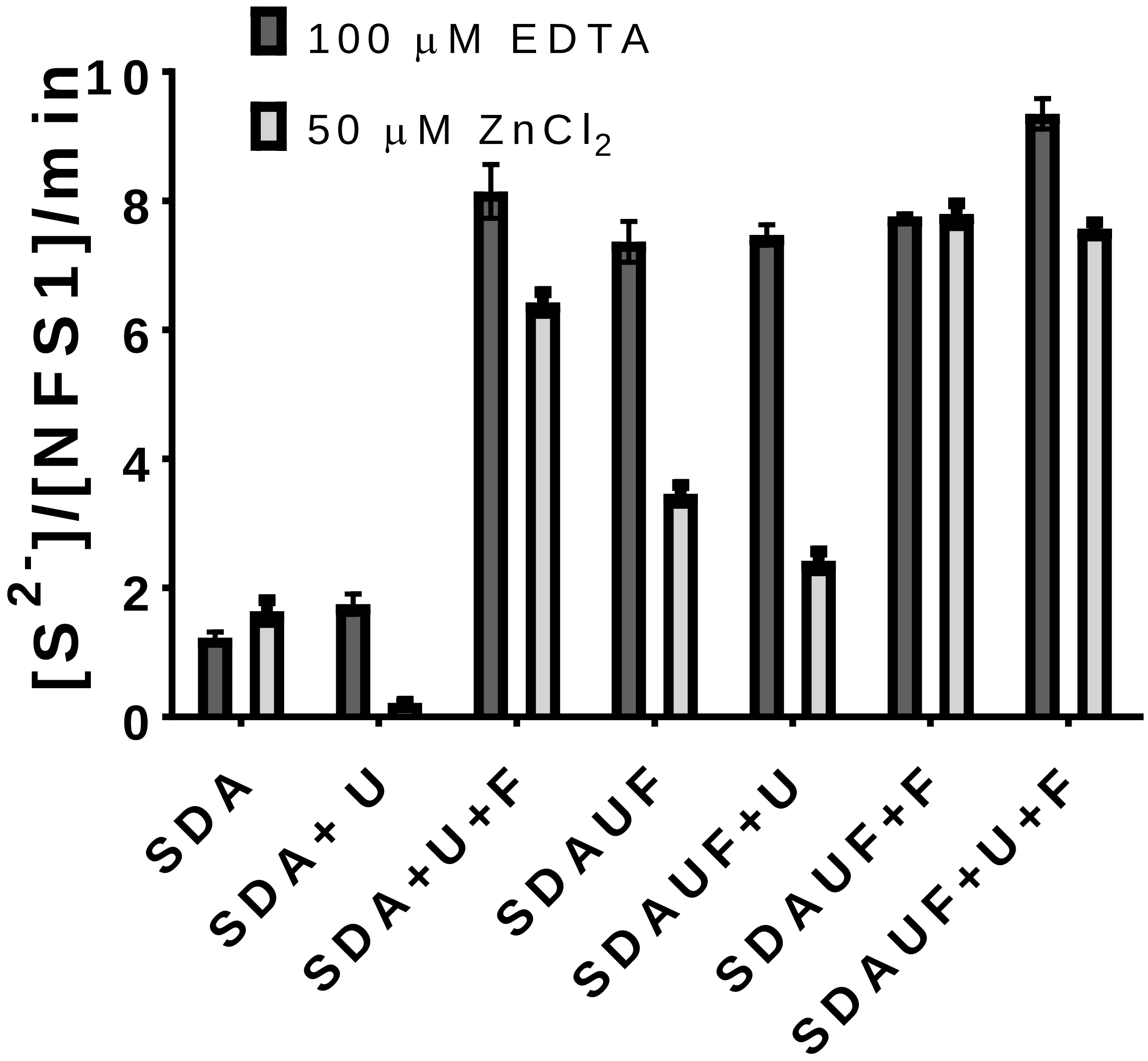

Supplementary Figure 3 Activity assay of purified SDAUF complex. NFS1 activity was measured by the methylene blue assay for individual complexes and combinations with and without Zn^{2+}. All complexes co-purified with E. coli ACP. The basal active form of complexes (SDA and SDA+U) could become fully activated when all 5 proteins were added (SDA+U+F, in a ratio of 1:5:5, respectively) regardless of the zinc inhibitor. The isolated complex (SDAUF) showed activity as great as when assayed with all components added separately, indicating that an activated complex was isolated in purification. When zinc was added to the assay, there was still some inhibition for the SDAUF complex, most likely due to FXN not staying fully bound to the complex. Adding additional ISCU (SDAUF+U) further reduced NFS1 activity, as ISCU is the target for zinc inhibition. The addition of more FXN (SDAUF+F or SDAUF+U+F) was able to overcome the zinc inhibition completely. Error bars indicate the standard deviation from technical replicates of n=3. Source data are provided as a Source Data file.

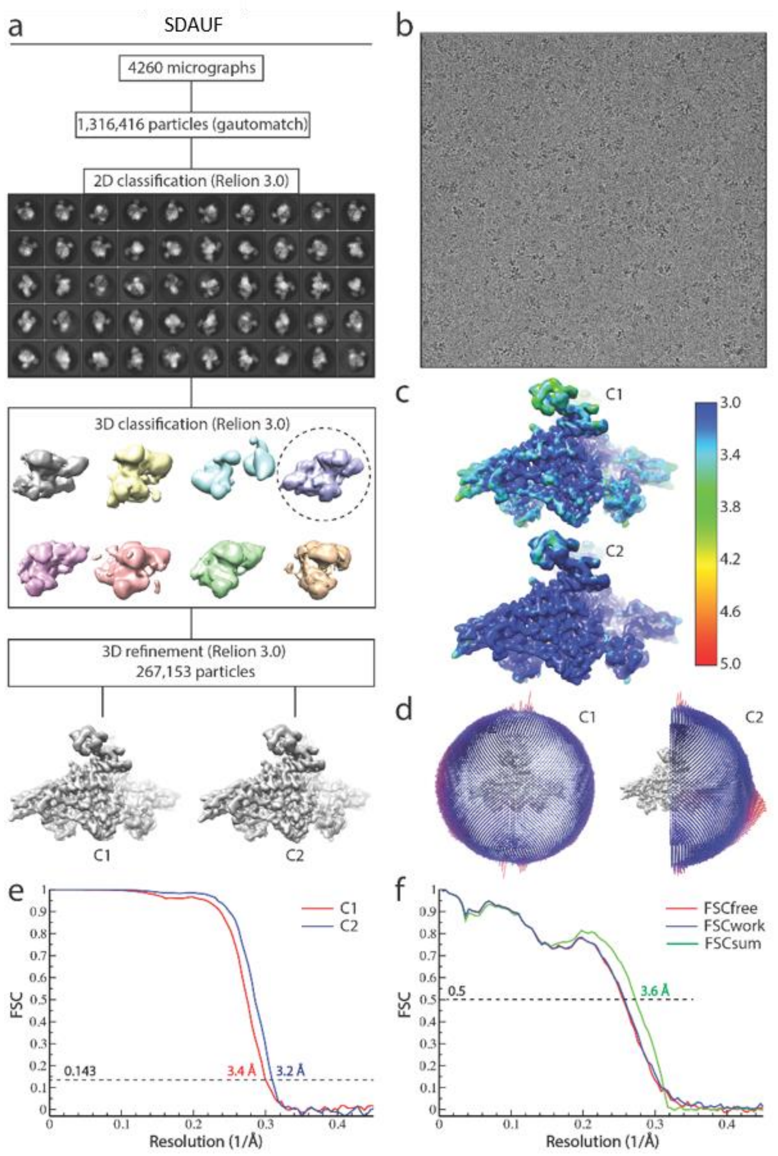

Supplementary Figure 4 Cryo-EM analysis of SDAUF complex. a. Flow chart of the cryo-EM data processing procedure. Details can be found in the Methods. **b.** A representative cryo-EM micrograph. **c.** Local resolution of the maps estimated using the ResMap program and colored as indicated. **d.** Angular orientation distribution of the particles used in the final reconstruction. The particle distribution is indicated by different color shades. **e**. Gold standard FSC curves of the structures with or without imposing C2 symmetry. **f.** Model validation. Comparison of the FSC curves between model and half map 1 (work), model and half map 2 (free), and model and full map are plotted in blue, red, and green, respectively.

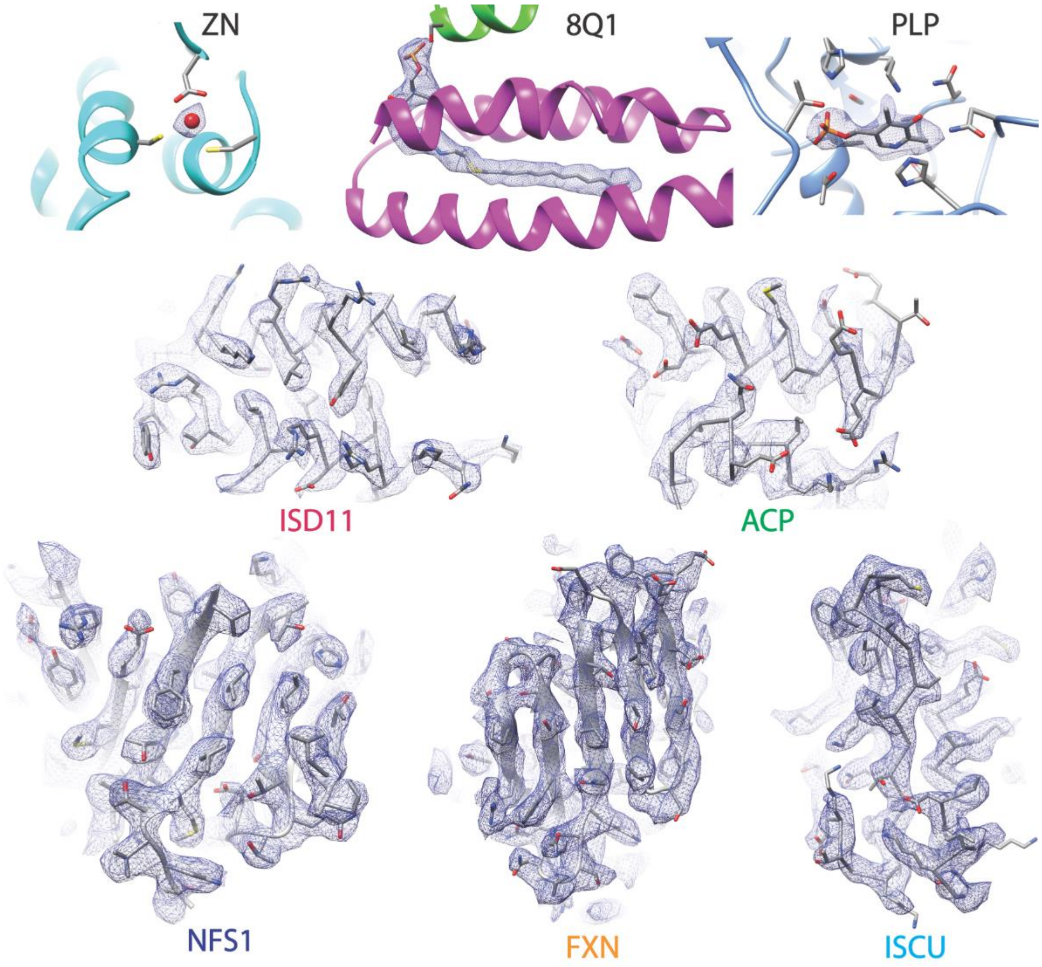

Supplementary Figure 5 Fit of the model to the density. Residues from SDAUF complex (in stick representation) and surrounding electron density maps are shown for exemplary regions. Cryo-EM density is displayed at the contour level 5-6.5 σ around the atoms. Subunits, ligands and maps are specified. Regions with best resolution include NFS1, ISCU, and FXN as well as ISD11. Poorest map quality was observed in the ACP regions.

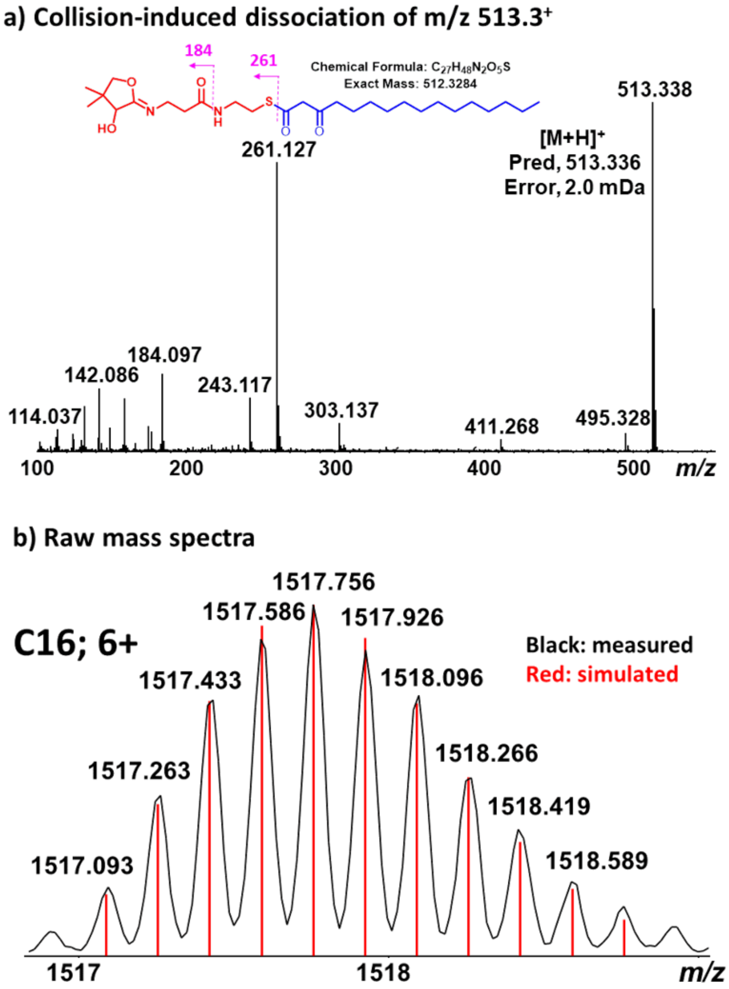

Supplementary Figure 6 LC-MS and MS^3 analysis. **a.** Using 3-ketoacyl-ACP (C16) as a representative example to show MS/MS/MS spectrum for the ejected 4'-PPT conjugated acyl-chains with both parent and fragment ions detected by ppm mass accuracy. Insert shows the proposed structure and fragmentation pathways. **b.** Measured isotopic distribution (black) of the 6+ ion of 3-ketoacyl-ACP (C16) agreed very well with the simulated one (red) by both relative abundances and ppm mass accuracy for all the detectable isotopes strongly supporting the proposed structure.

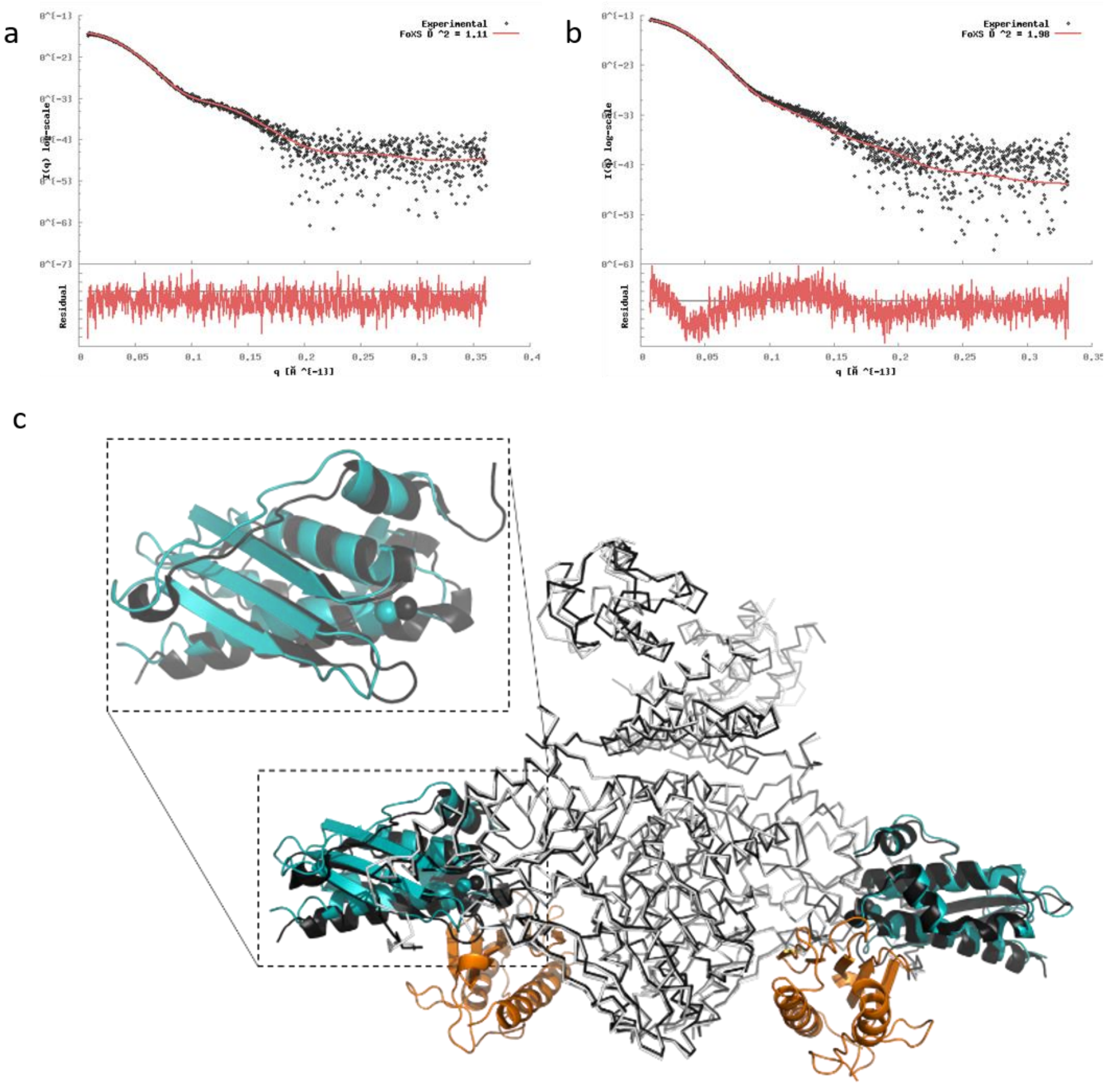

Supplementary Figure 7 SAXS analysis and structural superposition of complexes. a. Scattering data from a sample of SDAU was collected (black points) and fit to the theoretical SAXS profile back-calculated from SDAU-Zn^{2+} structure (5WLW)(red line) with χ^2=1.11. **b.** Scattering data from a sample of SDAUF used in cryo-EM was collected (black points) and fit to the theoretical SAXS profile back-calculated from the SDAUF-Zn^{2+} cryo-EM structure (red line) with χ^2=1.98. **c**. Superimposition of Cα chains from SDAUF-Zn^{2+} structure (this study; NFS1, ISD11, and ACP in white, ISCU in cyan, and FXN in orange) with crystal structures of SDAU-Zn^{2+} (5WLW; all subunits in black) showing a displacement of ISCU within the complex core upon FXN binding. Source data for panels a and b are provided as a Source Data file.

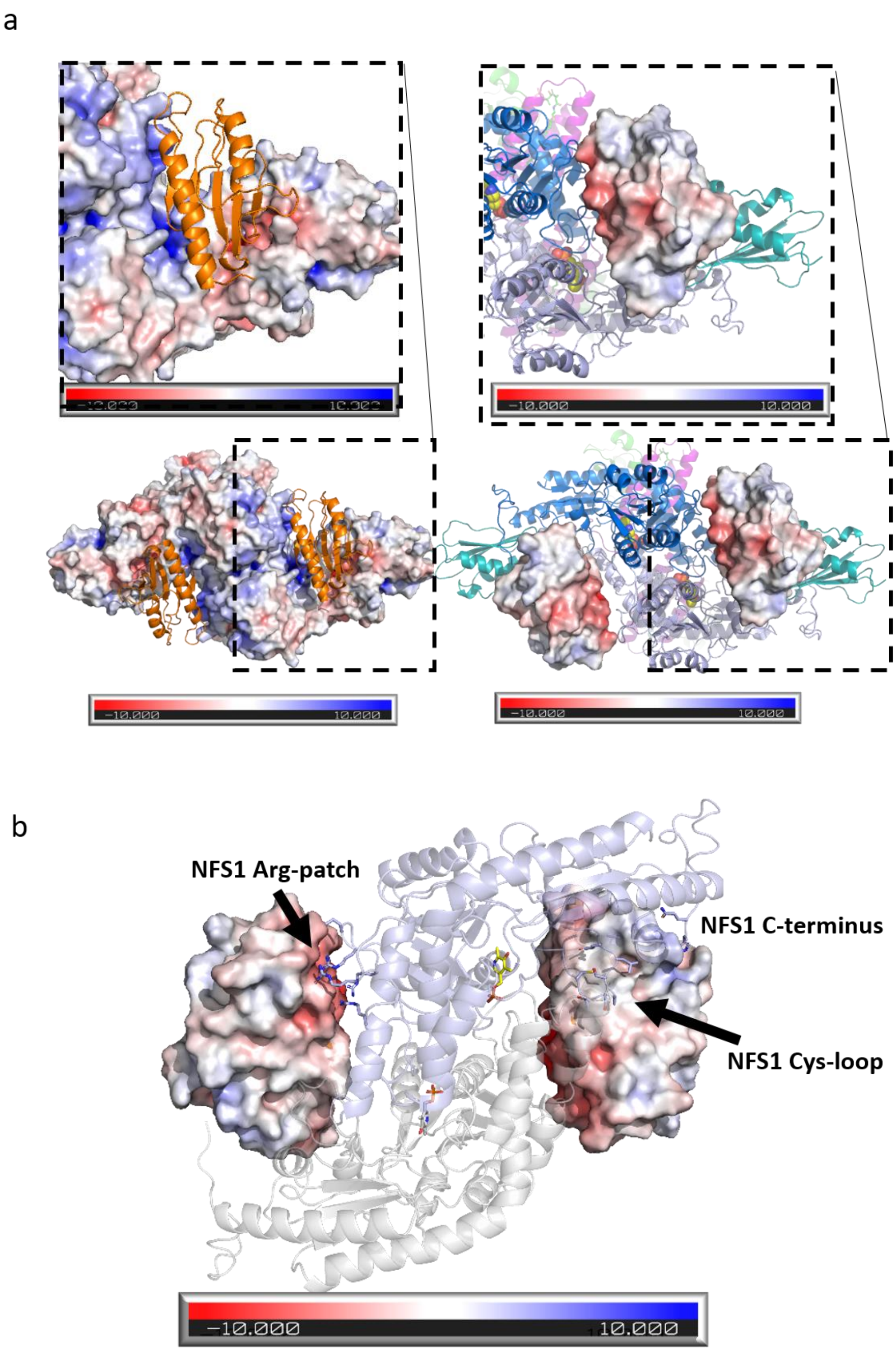

Supplementary Figure 8 Each FXN subunit interacts with both NFS1 subunits. a. Left: Surface representation of SDAU (from the SDAUF-Zn^{2+} structure), colored by electrostatic potential, shows a positively charged surface where FXN (orange cartoon) interacts. Right: Surface representation of FXN shows its negative surface that interacts with the positively charged surface on NFS1 (blue and slate cartoon). **b**. View down the 2-fold axis of NFS1 homodimer (slate/gray cartoon), showing interactions with both FXN protomers (surface representation) of the complex per NFS1. Each NFS1 uses its Cys-loop and C-terminus to contact one FXN protomer, and uses its Arg-patch on the other side of NFS1 to contact the other FXN protomer.

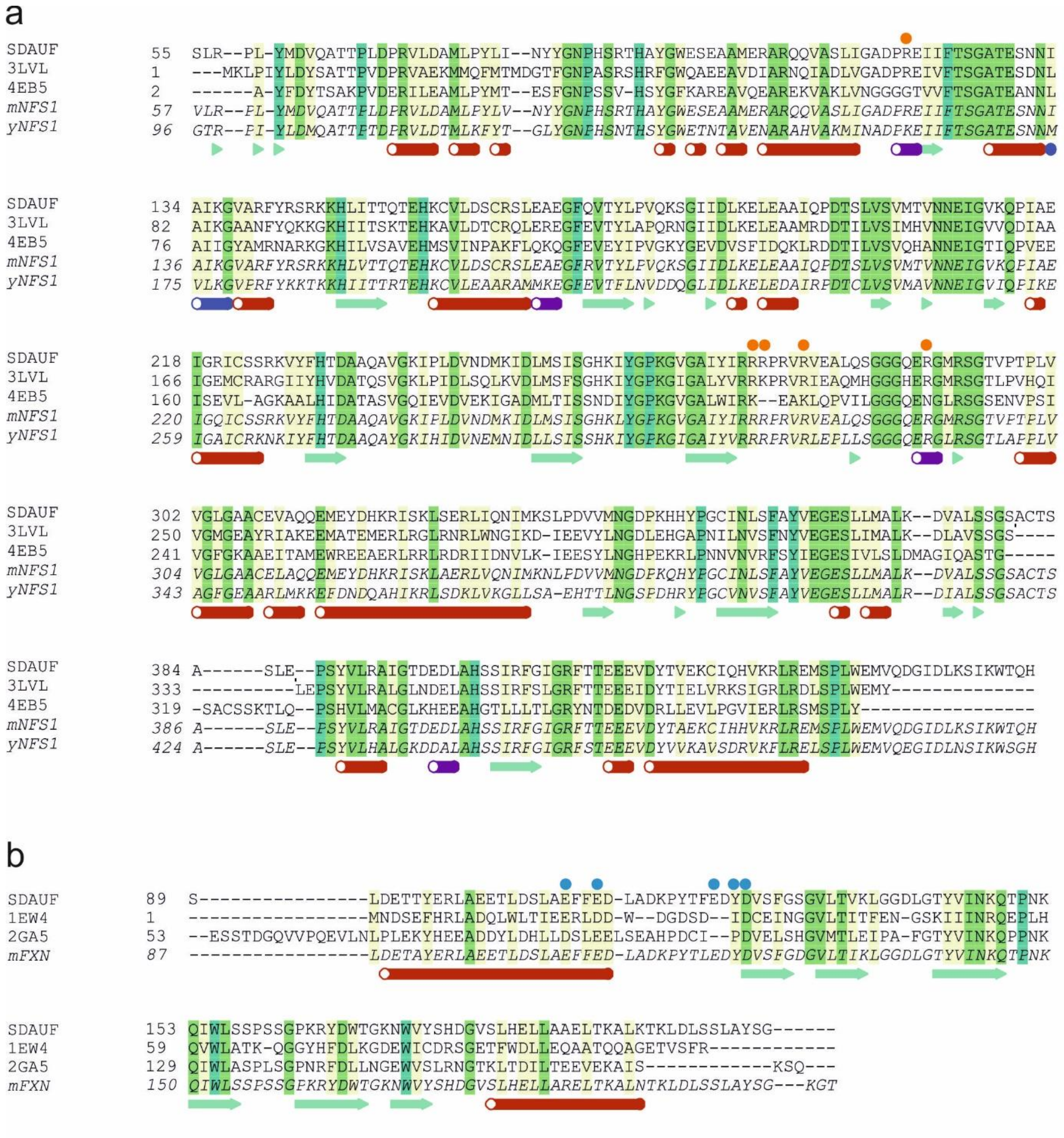

Supplementary Figure 9 Structure-based sequence alignment of FXN and NFS1. a. Sequence alignment of NFS1 orthologues, based on structural superposition of human SDAUF (this study) with *E. coli* IscS (PDB code 3LVL) and *Archaeoglobus fulgidus* IscS (4EB5). Primary sequences of mouse and yeast NFS1 (Uniprot codes Q9Z1J3 and P25374) are appended to this alignment. Orange dots above sequences denote human NFS1 residues forming potential hydrogen bonds/salt bridges with FXN, based on the SDAUF structure. **b**. Sequence alignment of FXN orthologues based on structural superposition of human SDAUF (this study) with *E.coli* CyaY (PDB code 1EW4) and yeast Yfh1 (2GA5). The primary sequence of mouse FXN (Uniprot code O35943) is appended to this alignment. Blue dots above the sequences denote human FXN residues forming potential hydrogen bonds/salt bridges with NFS1, based on the SDAUF structure. In both panels, secondary structure elements of NFS1 (a) and FXN (b) from SDAUF structure (α-helices, red; 3_{10} helices, purple; β-strands, green arrows) are shown below sequences.

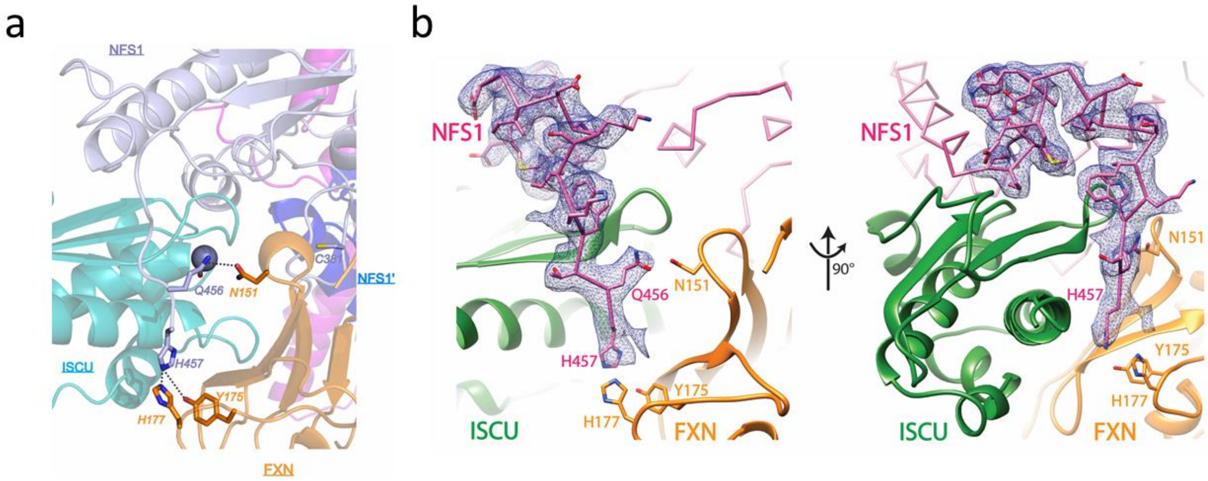

Supplementary Figure 10 Structural view of C-terminus of NFS1. a. NFS1 C-terminus (slate) wraps around ISCU (cyan), with the NFS1 terminal residues (Gln456 and His457; slate sticks) interacting directly with FXN Asn151, Tyr175 and His177 (orange sticks). **b.** Cryo EM density (contoured at 3σ) around the C-terminus of NFS1 was shown as blue mesh, in orthogonal views.
